# Life Review Intervention Delivered by Caregivers of Persons Living with Dementia Improves Depression

**DOI:** 10.1093/geroni/igaf122.1839

**Published:** 2025-12-31

**Authors:** Christina Miyawaki, Angela McClellan, Erin Bouldin, Cheryl Brohard, Mark Kunik

**Affiliations:** University of Houston, Houston, Texas, United States; University of Houston, Houston, Texas, United States; University of Utah, Salt Lake City, Utah, United States; University of Houston, Houston, Texas, United States; Baylor College of Medicine, Houston, Texas, United States

## Abstract

Approximately 40% of older Americans (≥65 years) living with dementia experience depression, yet behavioral interventions remain scarce. We developed a 6-week intervention, Caregiver-Provided Life Review (C-PLR) for people with dementia and depression and their family caregivers. Life review is an evidence-based depression intervention, in which patients retell their life stories to therapists chronologically. We trained caregivers in life review interview skills virtually, and caregivers conducted life reviews with their loved ones at home. This paper reports on the intervention outcomes and C-PLR’s acceptability and feasibility for participants. We used a mixed-methods design with 45 caregiver-care recipient dyads (N = 90). We measured care recipients’ depression (primary outcome), life satisfaction, caregiving rewards, caregiver burden, and dyads’ relationship quality (secondary outcomes) quantitatively. We asked about caregivers’ experiences conducting life reviews qualitatively. Caregivers were 58 years old on average, married, college-educated, working, female, and in good/excellent health while care recipients were 81 years old, widowed, retired, female, and in poor/fair health. Care recipients’ depression significantly improved (p < 0.001), as did caregiving rewards (p = 0.029), and relationship quality (p = 0.041) without affecting caregiver burden (p = 0.519). Caregivers supported the quantitative results in their interviews and confirmed the feasibility of virtual training and confidence in conducting life reviews. Training family caregivers to conduct life reviews may be an acceptable and cost-effective way to improve depression among people with dementia while C-PLR appeared to be a comprehensible and convenient depression intervention for their loved ones. Thus, C-PLR has the potential to reach a wide range of caregivers and people living with dementia.

